# Case Report: Malignant melanoma in a patient with Crohn’s disease treated with ustekinumab

**DOI:** 10.12688/f1000research.110356.2

**Published:** 2023-10-04

**Authors:** Georgios Axiaris, Alexandros Ioannou, Marina Papoutsaki, Leonidas Marinos, Michael Liontos, Spyridon Michopoulos, Evanthia Zampeli

**Affiliations:** 1 Gastroenterology Department, Alexandra General Hospital, Athens, Greece, 11528, Greece; 2 Dermatology Department, Syggros Hospital, Athens, Greece, 11528, Greece; 3 Pathology Department, Evangelismos Hospital, Atherns, Greece, 11528, Greece; 4 Oncology Department, Alexandra General Hospital, Athens, Greece, 11528, Greece

**Keywords:** Melanoma, IBD, Crohn’s disease, Ustekinumab

## Abstract

The cornerstone of inflammatory bowel disease (IBD) treatment is immunomodulators. IBD patients are at increased risk of intestinal and extraintestinal malignancy. Ustekinumab is a fully humanized monoclonal anti-IL12/23 antibody with a good safety profile. Malignancies of breast, colon, head and neck, kidney, prostate, thyroid, and non-melanoma skin cancer have been reported among patients who received ustekinumab. We report the case of a 42-year-old Crohn’s patient on long-term treatment with ustekinumab, who developed achromatic malignant melanoma. Crohn’s was diagnosed at the age of 15, with upper and lower gastrointestinal involvement and was initially treated with azathioprine (2mg/kg for 4 years) and infliximab (5mg/kg for 6 weeks). Due to ileal obstruction, the patient underwent stricturoplasty and received adalimumab (40mg every other week) for two years. He then discontinued therapy and a year later underwent right hemicolectomy. Adalimumab was reinstituted (40mg every other week) and the patient remained in clinical remission for two years. His overall exposure to adalimumab was four years. Ustekinumab was initiated due to a relapse and after 3 years, an incident of scalp itching led to the diagnosis metastatic achromatic malignant melanoma bearing BRAF V600E mutation. He received targeted therapy with an initial good response. We aim to point out the risk of dermatologic malignancy in IBD patients on long-term immunosuppression and the lifelong and meticulous evaluation that is required.

## Introduction

Crohn’s disease is a chronic inflammatory disorder of unknown etiology. Patients with inflammatory bowel disease (IBD) are at a higher risk of developing colorectal cancer as well as extraintestinal malignancies, probably related to chronic inflammation as well as immunosuppression. ^1^^–^^4^ Malignancies associated with long-term suppression of the immune system in the IBD setting, include skin cancers, myelodysplastic syndromes, lymphomas, acute myeloid leukemia, and urinary tract cancers. ^5^ Melanoma is potentially aggressive, highly immunogenic, and strongly related to UV radiation exposure. An increased risk of melanoma has been observed in IBD patients, especially those exposed to tumor necrosis factor (TNF)-α antagonists in contrast to those receiving thiopurines who have an increased risk of non-melanotic skin cancers. ^6^^–^^8^ The risk for melanoma increases 1.5 to 2 times for those exposed to TNF-α antagonists. ^9^ Ustekinumab, a fully humanized interleukin (IL)-12/23 monoclonal antibody, targets the common p40 subunit shared by IL-12 and IL-23. The incidence of malignancies was comparable between ustekinumab-exposed psoriatic patients and the general population according to a study with three years follow up. ^10^ The reported malignancies share no particular pattern (colon, breast, kidney, thyroid, head and neck, prostate, and non-melanoma skin cancer). Achromatic malignant melanoma has not been reported so far. Data regarding ustekinumab and development of melanoma are scarce. ^11^ We report the case of a 42-year-old Crohn’s patient on long-term treatment with ustekinumab, who developed achromatic malignant melanoma.

## Case presentation

Α 42-year-old male patient with Crohn’s disease who was under treatment with ustekinumab developed achromatic malignant melanoma. The patient’s family history was negative both for malignancy and IBD. The patient is a current smoker with alcohol consumption of less than 10 units per week.

Crohn’s was diagnosed at the age of 15, with upper and lower gastrointestinal involvement (Montreal classification A1L3L4B2p). Specifically, the patient was diagnosed with a perianal abscess a few years after Crohn’s diagnosis and was managed with antibiotics. The perianal lesions responded well to Crohn’s treatment and remained inactive over the years. The patient was initially treated with azathioprine (100 mg/day for 4 years) and infliximab (5 mg/kg every 8 weeks). Infliximab was discontinued due to an allergic reaction during the third infusion. Whilst on azathioprine for four years, the patient was admitted to hospital due to ileal obstruction and was effectively treated with multiple stricturoplasties.

Postoperatively, the patient received adalimumab (40 mg every other week) for two years; he discontinued the treatment on his own accord. A year later the patient underwent right hemicolectomy due to obstruction; an anastomotic leak led to a temporary ileostomy. Adalimumab was reinstituted and the patient remained well for two years when peripheral polyneuropathy occurred. This was attributed to adalimumab, once other possible causes were excluded, leading to its discontinuation after an approximate overall exposure of four years. Moreover, endoscopy revealed a sigmoid stenosis and a third operation was performed for excision of colonic stenosis and restoration of bowel continuity. Six months after surgery, the patient presented with elevated inflammatory markers [calprotectin 676 mcg/g (normal value <50), C-reactive protein (CRP) 45 mg/L (normal value <5)]. Endoscopy revealed multiple large ulcers in the ileocolonic anastomosis and ileum corresponding to an endoscopic Rutgeerts score of i2b. Ustekinumab was initiated in January 2018 (initial dose of 390 mg intravenously according to patient’s weight and 90 mg subcutaneously every eight weeks thereafter).

**Figure 1.**
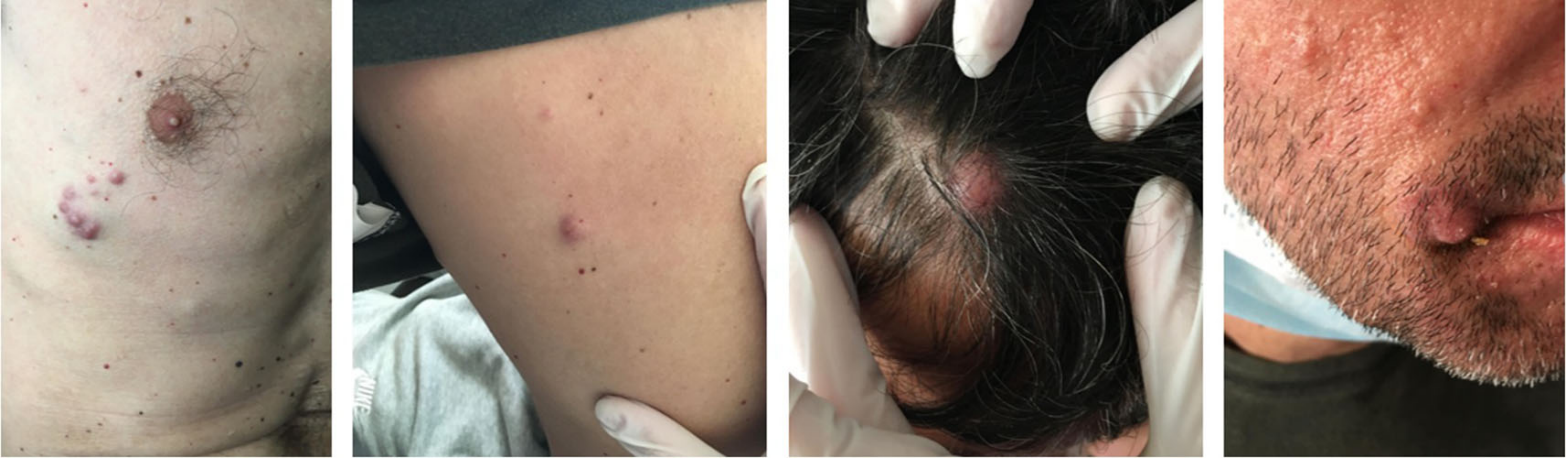
Non-melanotic subcutaneous nodules on the trunk, arms, scalp and face rapidly expanding to multiple sites.

**Figure 2.**
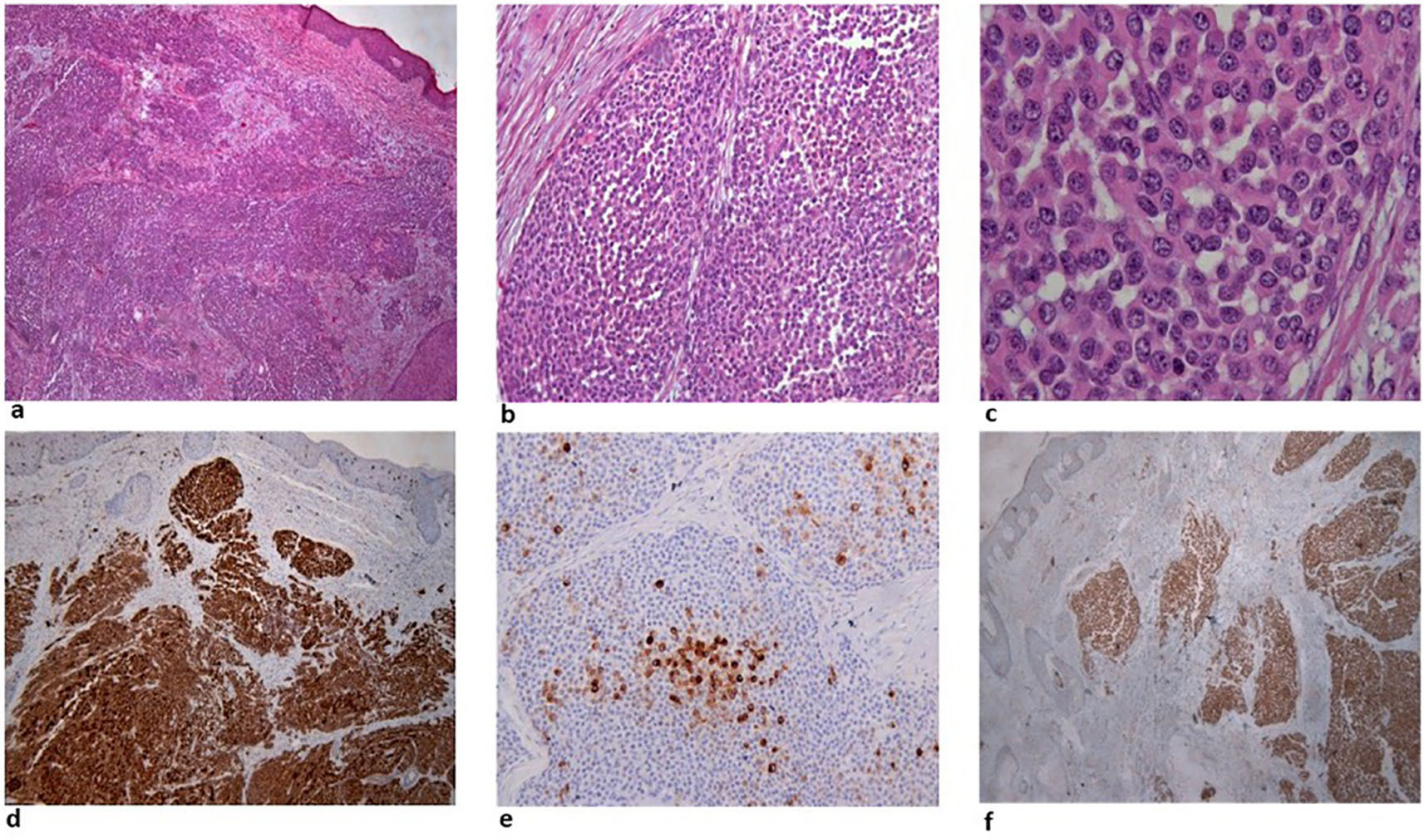
a: Extensive infiltration of the dermis by large aggregates of neoplastic cells. b: The neoplastic cells are large with eosinophilic cytoplasm and mostly rounded nuclei. c: Larger magnification showing details of the cytomorphology, focally increased mitotic activity and complete absence of melanin. d: Uniform staining of the malignant cells for S-100. e: Focal staining for HMB45. f: Strong and uniform staining with BRAF V600E (VE1) antibody.

Three years after initiation of ustekinumab, the patient complained about scalp itching. Clinical examination revealed a scratched bleeding mole on the scalp. Dermatologic assessment, which included full body inspection, was initially reassuring. One month later the patient complained about intense fatigue and presented with multiple, hard, subcutaneous nodules initially on the scalp and rapidly expanding on the trunk and arms ( Figure 1). A surgical biopsy was performed, and while awaiting histology, the patient presented to the emergency department with severe dyspnea. Computed tomography (CT) scans showed multiple, large, nodular lesions in the brain, lung, liver, pericardium, adrenal glands, kidneys and abdominal muscles, as well as multiple osteolytic lesions. Histology showed an extensive infiltration of the dermis with sparing of the epidermis, by large aggregates of neoplastic cells with variable amount of eosinophilic cytoplasm and mostly round nuclei with uneven chromatin distribution ( Figure 2a-c). Despite a thorough examination of all available sections and fields, no melanin pigment was found. Immunohistochemistry revealed strong and uniform staining for S-100 and BRAF V600E (VE1) and focal staining for HMB45 ( Figure 2d-f). The intensity of staining with the monoclonal BRAF V600E (VE1) antibody was graded as 3. ^11^

The patient was diagnosed with metastatic cutaneous melanoma bearing the BRAF V600E mutation according to immunohistochemical testing. The clinical status of the patient rapidly deteriorated with acute respiratory failure and acute kidney injury. He was therefore started on treatment with combined BRAF/MEK inhibition with dabrafenib (150 mg twice a day) and trametinib (2 mg daily) under the provisional approval of the regulatory authorities. Since day 3 of treatment, clinical improvement has been noted with improvement of dyspnea, renal function and mental status.

The patient tolerated treatment without any severe adverse events. Most of the nodular lesions started shrinking by day 5 of treatment and the patient was discharged from the hospital on day 20. At discharge, almost all nodular lesions on the skin and tongue disappeared and renal and lung function returned to normal. The patient continued combined BRAF/MEK inhibition. However, he gradually presented cognitive impairment and developed psychiatric symptomatology, which were attributed to persistent brain metastases.

## Discussion

We present a case of a patient with complicated Crohn’s disease, who underwent repeated surgeries and received prolonged immunosuppression with azathioprine, TNF-α antagonists and ustekinumab. The patient ultimately developed melanoma.

Ustekinumab (Stelara ^®^) is a fully human monoclonal antibody against the common p40 subunit of IL-12 and IL-23. According to existing evidence, IL-12 and IL-23 may influence the development and behavior of melanoma. In particular, murine models show that IL-23 influences the growth and progression of melanomas. Since human melanocytes and melanoma cells express IL-23 receptors, the neutralization of IL-23 may potentially increase the susceptibility of melanoma development. ^12^ IL-12 deficiency promotes photo-carcinogenesis and angiogenesis in UV radiation-induced skin cancers, ^13^^,^^14^ thus providing a possible explanation of how anti-IL-12/IL-23 therapy may exacerbate the risk for UV radiation-induced skin malignancies. ^15^ IL-12 has demonstrated anti-tumor activity via induction of interferon (IFN) γ production from natural killer cells, CD4+ and CD8+ T cells, thus modifying the tumor microenvironment. ^16^ Data from the pivotal UNITI studies and real-world data are reassuring regarding the safety of ustekinumab. ^17^ The data for 2574 patients included in six phase 2/3 studies corroborated the favorable safety profile of ustekinumab. ^18^

To date, no clear link between ustekinumab and cutaneous carcinogenesis has been demonstrated. Regarding squamous cell carcinoma and ustekinumab, a few case reports in patients with psoriasis have been published. ^19^ A case of a patient with psoriasis vulgaris and a history of melanoma, who received ustekinumab for seven consecutive years without relapse of melanoma, was reported. ^20^ A 53-year-old female with severe psoriatic arthritis and a history of metastatic melanoma received sequential therapy with secukinumab and ustekinumab (for nearly two years) and no melanoma relapse or progression was recorded. ^21^

There is insufficient evidence regarding the oncogenic potential of ustekinumab. It is difficult to establish an association between treatment with ustekinumab and melanoma in our patient since, on one hand, Crohn’s increases the risk for malignancy and on the other, our patient was exposed to multiple immunosuppressive treatments over the years. Although a strong association cannot be established, this case is worth reporting as the prolonged exposure to ustekinumab justifies the hypothesis of a possible connection. The management of patients who develop malignancy while on immunosuppressives is quite demanding as immune suppression is associated with worse survival. ^22^ By means of this case, we would like to point out the risk of malignancy for IBD patients on long-term immunosuppression and the strict and meticulous follow-up that is required. Emphatically, a prophylactic total body dermatologic assessment should not be neglected in IBD patients.

## Data availability

All data underlying the results are available as part of the article and no additional source data are required.

## Consent

Written informed consent for publication of the patient’s clinical details and clinical images was obtained from the relative and guardian of the patient. The patient was cognitively and psychiatrically unstable meaning he was unable to give consent to publish.
