# Supplementary material for: Case Report: Malignant melanoma in a patient with Crohn’s disease treated with ustekinumab
Source: F1000Res. 2023 Oct 4;11:424. Originally published 2022 Apr 14. [Version 2] doi: 10.12688/f1000research.110356.2 (PMC10589619; doi:10.12688/f1000research.110356.2)
Supplement: Peer review history

# Reviewer report: Reviewer response for version 2

**DOI:** 10.5256/f1000research.156888.r212109

**Published:** 2023-10-20

**Authors:** Sophie Vieujean

**Affiliations:** 1 University Hospital CHU of Liège, Liège, Belgium

The revisions are appropriate for me.

Are enough details provided of any physical examination and diagnostic tests, treatment given and outcomes?

Partly

Is the case presented with sufficient detail to be useful for other practitioners?

Partly

Is sufficient discussion included of the importance of the findings and their relevance to future understanding of disease processes, diagnosis or treatment?

Partly

Is the background of the case’s history and progression described in sufficient detail?

Yes

Reviewer Expertise:

NA

I confirm that I have read this submission and believe that I have an appropriate level of expertise to confirm that it is of an acceptable scientific standard.

# Reviewer report: Reviewer response for version 1

**DOI:** 10.5256/f1000research.121953.r188078

**Published:** 2023-08-03

**Authors:** Sophie Vieujean

**Affiliations:** 1 University Hospital CHU of Liège, Liège, Belgium

- Perianal status not clear
- The presence of brain metastases should be explained earlier
- "According to existing evidence, IL-12 and IL-23 influence the development and behavior of melanoma." This statement should be formulated with more caution because, as mentioned by authors a bit latter "no clear link between ustekinumab and cutaneous carcinogenesis has been demonstrated" 

Are enough details provided of any physical examination and diagnostic tests, treatment given and outcomes?

Partly

Is the case presented with sufficient detail to be useful for other practitioners?

Partly

Is sufficient discussion included of the importance of the findings and their relevance to future understanding of disease processes, diagnosis or treatment?

Partly

Is the background of the case’s history and progression described in sufficient detail?

Yes

Reviewer Expertise:

Inflammatory bowel diseases

I confirm that I have read this submission and believe that I have an appropriate level of expertise to confirm that it is of an acceptable scientific standard, however I have significant reservations, as outlined above.

# Response

**Published:** 2023-09-30

**Authors:** ALEXANDROS IOANNOU

**Affiliations:** Alexandra Regional General HospitalDepartment of Gastroenterology, Greece

Dear Dr. **Vieujean**

 We would like to thank you for reviewing our manuscript. Here are the answers to your valuable comments:

  

1. Regarding the perianal status of the patient: *“Specifically, the patient was diagnosed with a perianal abscess a few years after Crohn’s diagnosis and was managed with antibiotics.  The perianal lesions responded well to Crohn’s treatment and remained inactive over the years.”*
2. The brain metastases were diagnosed once the patient was admitted to the hospital due to dyspnea and deranged mental status. Before that, the evaluation only regarded the skin manifestations, as there were no other symptoms. Whist awaiting for the histology results, the patient developed acute respiratory distress and it was then that he underwent CT scans that revealed the lesions in other organs.    
3. We revised the relevant sentence following your kind suggestion: “According to existing evidence, IL-12 and IL-23 **may **influence the development and behavior of melanoma”

# Reviewer report: Reviewer response for version 1

**DOI:** 10.5256/f1000research.121953.r148374

**Published:** 2022-09-05

**Authors:** Giovanni Marasco

**Affiliations:** 1 Department of Medical and Surgical Sciences, University of Bologna, Bologna, Italy

The report is well-written and exhaustive. The case reports on a new connection between ustekinumab, crohn's disease and melanoma. It can be useful for clinicians to increase the awareness of this possible complication. Well done. My only suggestion is to add aa table summarizing the updated effect of ustekinumab regarding cancer development

Are enough details provided of any physical examination and diagnostic tests, treatment given and outcomes?

Yes

Is the case presented with sufficient detail to be useful for other practitioners?

Yes

Is sufficient discussion included of the importance of the findings and their relevance to future understanding of disease processes, diagnosis or treatment?

Yes

Is the background of the case’s history and progression described in sufficient detail?

Yes

Reviewer Expertise:

Microbiota, GI Immunology

I confirm that I have read this submission and believe that I have an appropriate level of expertise to confirm that it is of an acceptable scientific standard.
